# An observational study on the effect of premature ventricular complex burden on long-term outcome: Erratum

**DOI:** 10.1097/MD.0000000000007119

**Published:** 2017-06-02

In the article, “An observational study on the effect of premature ventricular complex burden on long-term outcome”,^[1]^ which appeared in Volume 96, Issue 1 of *Medicine*, affiliation b should have appeared as “Institute of Clinical Medicine and Cardiovascular Research Center, National Yang-Ming University, Taipei.”
